# Cultural and Social Bias Leading to Prenatal Sex Selection: India Perspective

**DOI:** 10.3389/fgwh.2022.903930

**Published:** 2022-06-13

**Authors:** Nayan Chakravarty, Vandana Dabla, Moni Sagar, Sharmila Neogi, Mridu Markan, Mehak Segan, Shilpi Agnani, Pooja Kapahi, Sourav Neogi

**Affiliations:** ^1^USAID's MOMENTUM Country and Global Leadership: India-Yash, Jhpiego, New Delhi, India; ^2^USAID, New Delhi, India

**Keywords:** missing female births, sex ratio at birth (SRB), gender bias, son preference, prenatal sex selection

## Abstract

Globally, 23.1 million missing female births have been documented, resulting in an imbalanced sex ratio at birth (SRB) between the late 1990s and 2017, with India accounting for almost half of this missing women population. While the country is progressively taking measures to enhance women's position in society and implementing policies toward augmenting the value of a girl child, some deeply rooted cultural and social beliefs propel a strong son preference, resulting in active daughter discrimination. The continuance of patriarchal norms and inequitable gender roles, resulting in son preference, fertility decrease, and reduction in preferred family sizes, and technical breakthroughs that allow for the identification of the sex of the fetus, is all connected to distortions in the sex ratio at birth. Son preference is a well-documented phenomenon in India, and its implications for skewed gender ratios, female feticide, and higher child mortality rates for girls have piqued researchers' and policymakers' interest. The fundamental factors of son preference as an ideology are less widely investigated. With this objective, an extensive secondary review was conducted of the socio-cultural norms and biases leading to increased prenatal sex selection in India despite the laws against it. The study findings suggest that it is imperative to emphasize the necessity for consistent and collective efforts from all stakeholders: changing the social perception of the value of girls requires collective effort and the equal participation of all stakeholders, including civil society organizations and the local community.

## Introduction

Globally, 23.1 million missing female births have been documented, resulting in an imbalanced sex ratio at birth (SRB) between the late 1990s and 2017, with India accounting for almost half of this missing women population at 10.6 million. The United Nations defines gender-based sex selection as one of the harmful practices likely causing male-biased sex ratios in countries in Asia, Africa, and Eastern Europe rooted in the complex web of socio-economic and cultural variables. The biologically typical sex ratio is 102–106 males per 100 females at birth. However, the higher-than-normal ratios as high as 130 males or above are becoming a cause of concern in many South Asian, East Asian, and Central Asian countries (1–3). In 1990, Amartya Sen, The Nobel Laureate declared that over 100 million women had gone missing in Asia (4). According to the state of world population report 2020 released by the United Nations Population Fund, 460,000 girls were missing at birth each year in India between 2013 and 2017, while 0.59 million female births were missing between 2015 and 2020 due to prenatal sex selective elimination of a girl child. Although women have a biological survival advantage over men, a combination of “mortality and natality inequality” has adversely affected the child sex ratio (CSR) and SRB (5, 6).

As per India's fifth round of the National Family Health Survey (NFHS 5) held between 2019 and 2021, the sex ratio of the population (females per 1,000 males) was estimated as 1,020, clocking a female majority for the first time (7). This celebration has been overshadowed by an alarmingly low SRB of 929, indicating continued sex selection at birth. In patriarchal societies like India, there are hierarchical gender relations, local female agency, and a strong desire for boys, which result in selective abortion of female fetuses occurring within this context (8, 9). This observation is further supported by previous studies, identifying increased mortality rates among girls and women as the primary cause of uneven sex ratios in the populations of several Asian countries (10–13). Thus, a stronger preference for sons over daughters and gender discrimination lead to higher female mortality (14). The Sustainable development goal number 5 given by the United Nations, promises to achieve gender equality and empower all women and girls. It is every girl's fundamental right to be given an equal opportunity to be born into the world and thrive along with her male counterparts. Every girl's fundamental right is to be given equal opportunity to be born into the world and thrive along with her male counterparts. Still, our daughters in India face more discrimination than sons (15, 16) due to socioeconomic, cultural, and historical factors (17, 18).

Son preference presents itself in various ways in strongly male-dominated civilizations: sons are seen to have higher cultural and economic value, and the way this manifests can range from differential allocation of household resources, medical care, and neglect of girl children to female infanticide. Inheritance and land rights are passed down through male heirs, aging parents rely on male support in the absence of national security plans, and married daughters are not allowed to look after their birth family by marital family in-laws and husbands. On the other hand, women requiring dowries in marriage and leaving the biological family upon marriage make daughters to be perceived as an economic burden by the family. Furthermore, some religious and cultural customs are only performed by a son, such as rituals upon parents' death in some religious societies (14, 19).

The fundamental factors of son preference as an ideology are less widely investigated. With this objective, a review was conducted of the socio-cultural norms and biases for a strong son preference, leading to increased prenatal sex selection in India. The findings of this study shall be helpful for public health workers, health professionals, and civil society organizations involved in increasing the value of a girl child. This shall further inform policymakers who are considering the issue of prenatal sex determination toward better policy decisions.

## Prenatal Sex Selection and Sex Ratio at Birth

Sex selection is a complex issue wherein stakeholders may have conflicting interests and rights. It encompasses individual women and their partners who engage in sex selection and their family members, such as in-laws, health professionals/service providers, and society, including men who cannot find brides and women who may face indirect consequences of forced marriages or trafficking. Cultural beliefs also propel active daughter discrimination. Global evidence also shows that social norms related to higher male authority and female obedience strongly correlate with the levels of gender-based violence (GBV). One of the most blatant types of gender-based violence is the decision to continue or terminate a pregnancy based on the sex of the baby. This adds to the burden of other forms of GBV on women and young girls. National Crime Records Bureau of India categorizes the crimes targeted at women under three major groups, namely, intrafamily violence, interpersonal violence, and societal/general violence. Some examples under these categories are dowry deaths, abetment of suicide for women, causing miscarriage without the consent of the women, etc. (20). Restricting her healthcare, controlling her finances, being coerced into sexual relations by an intimate partner, or general household maltreatment are just the tip of the iceberg of the different types of abuse prevalent in Indian society.

There is no official documentation of prenatal sex selection, but the number of incidents can be approximated indirectly using the deviation of the observed SRB from the natural level (21). In recent decades, India's SRB has shifted toward masculinity. Decreased fertility and the availability of and access to sonographic scanning during pregnancy contribute to the sex ratio imbalance. The NFHS 5 reveals that Uttar Pradesh, Haryana, Punjab, Rajasthan, Bihar, Delhi, Jharkhand, Andhra Pradesh, Tamil Nadu, Odisha, Maharashtra are the major states with the low SRB. A sharp decline in India's CSR is also evident in the three decades from 1981 to 2011 when it fell from 962 to 919 (22). Therefore, understanding the underlying cause of sex selection is crucial to interpreting India's sex ratio dynamics.

**Figure 1:**
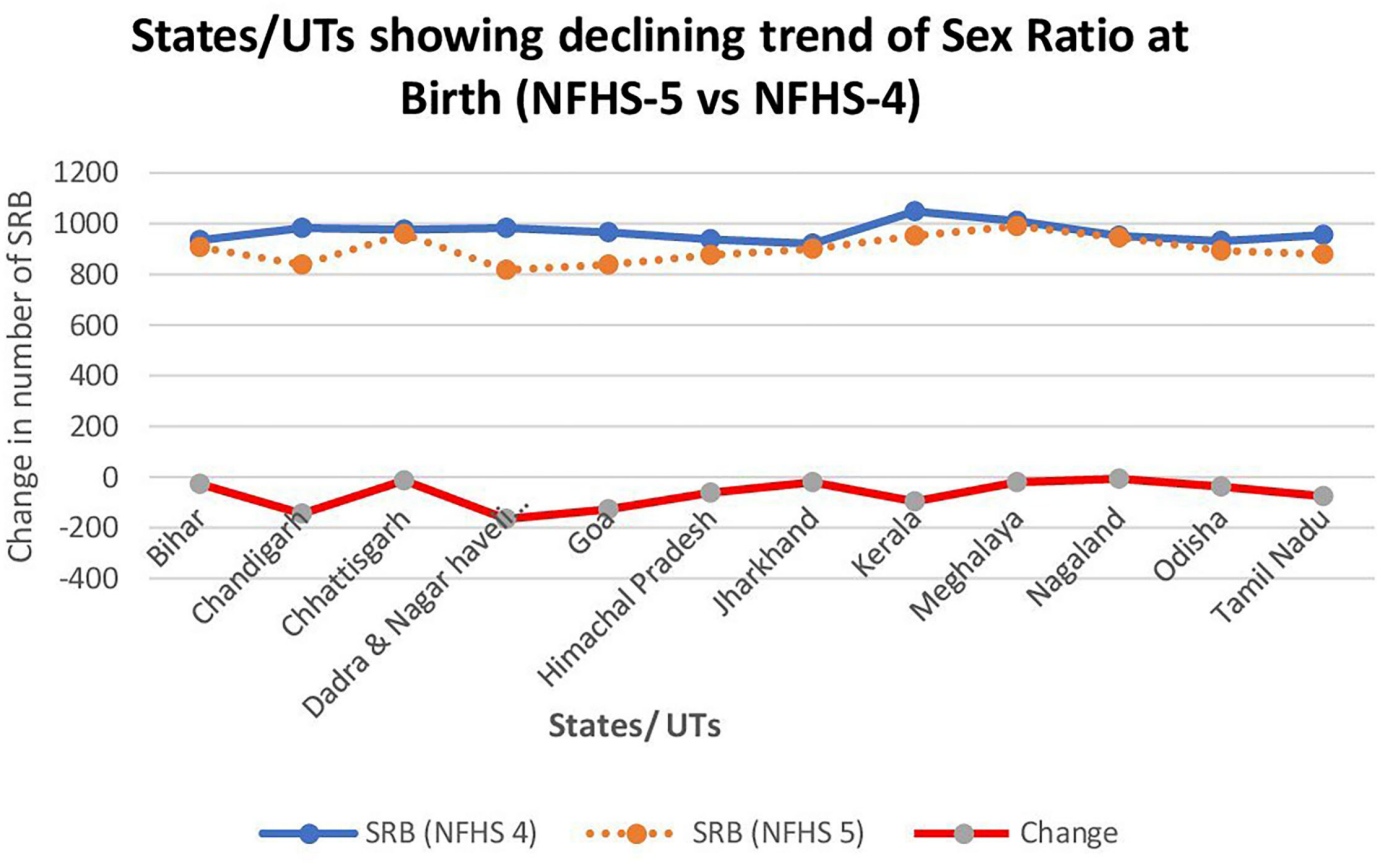
States/UTs having declined in Sex Ratio at Birth as per NFHS 5 (2019–2021) vs. NFHS 4 (2015–16).

However, India's SRB has shown improvement from 919 in NFHS-4 to 929 in NFHS-5, an increase of 10 points. The national capital territory of Delhi has improved its CSR from 812 to 923, an increase of 111 points. In addition, the SRB in Ladakh, Lakshadweep, Sikkim, and Puducherry has also increased by more than 100 points. However, some states that registered a lowered SRB in NFHS 5 compared to NFHS 4 are Maharashtra, Chhattisgarh, Himanchal, Tamil Nadu, and Kerala (Figure 1).

## Cultural and Social Norms: Essential Elements of Prenatal Sex Selection in India

In India, a standard way of referring to a girl born in a family is *ParayaDhan*, meaning “someone else's property.” Such colloquial terms are symptomatic of the devaluation of women in general. In some sections of the community, women do not have total autonomy over their lives because they move from their parents' house to their marital home; they rarely have any say in their decisions (21). Furthermore, females spend two to ten times more time on unpaid care tasks than males worldwide, like young girls are made to miss out on school to take care of their younger siblings. Due to gendered cultural norms that see unpaid care labor as a female prerogative, women from all regions, socioeconomic categories, and cultures spend a large number of their days meeting the expectations of their domestic and reproductive chores. This, along with their paid work, creates a “double load” of work for women (23).

Most countries worldwide have patriarchal traditions, but they appear to be particularly ingrained in significant sections of the Indian subcontinent. Patriarchal rules and views affect marriage arrangements, lineage regulations, and female seclusion. Social gender norms are also influenced by traditional labor distribution (24). Due to this sociocultural construct, girls are denied the right to attend school and work outside their natal homes (25).

Additionally, young girls are married and have no right of inheritance to property in many male-dominated communities. Early marriage and motherhood can cause interruptions in education, social isolation, and a lack of career and training options for girls, leaving limited employment opportunities for them and hence making them financially dependent on their husbands and in-laws (5).

Masculine hegemony in all aspects of social structure results in a male-dominated society where women are seen as only a way for men to reproduce. Sons are frequently seen as a hopeful source of stability and a way to better one's social position by women in such settings (26). Social and cultural norms about gender roles, family structures, and intrafamily violence are determined by ancient socioeconomic conditions, which remain even when the underlying conditions alter (27). Women's low negotiating power inside the marriage, dowry system, inheritance, and land rights make them more vulnerable and devalued as an asset. They are considered less “profitable” and produce a lesser “return on investment.” Such marriage patterns and housing arrangements are further linked to less preference for the girl child in India (24, 28).

Moreover, the necessity for male offspring characterizes patrilineal family systems. In Indian society, sons carry on the family name, property ownership, and key family traditions. Family assets are passed down through the male line, whereas women may be granted movable property as a dowry. Furthermore, patrilineality and patrilocality go hand in hand. Married sons are supposed to live with their parents following marriage, whereas married daughters are expected to leave home (29–31).

## Daughter Neglect, Female Status, and Autonomy

**Figure 2:**
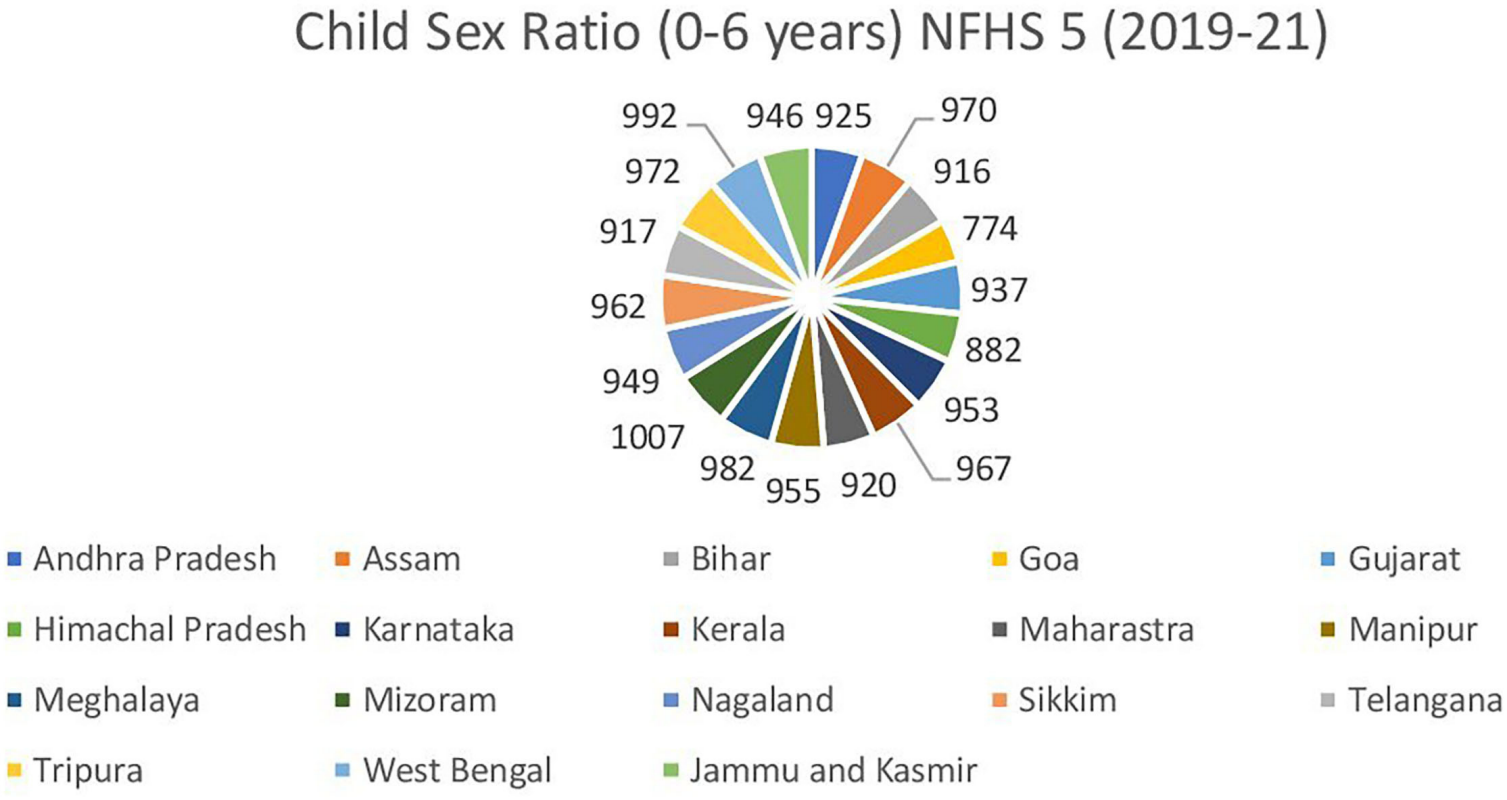
Child sex ratio (0–6 years) NFHS-5 (2019–21).

While feticide resulting from prenatal sex selection occurs before birth, discriminatory treatment of daughters in nutrition, immunization, education, and other basic needs begins in early life. Boys are given better care than their female siblings. According to demographic indicators, such as an imbalanced child sex ratio (see Figure 2) and increased female child mortality (32–35), postnatal neglect of a girl child is still a problem in the twenty-first century.

According to several research studies, sex selection against females is more common at higher maternal parity when only girls have previously been born, implying that prenatal sex selection is used to ensure that at least one son is born. As a result, as fertility declines, parents' proclivity to revert to prenatal sex selection to secure a male progeny may increase, increasing gender bias (36, 37).

## Technological Advancement

The availability of modern techniques to determine the sex of the fetus has also provided its users with an easy option of gender-based selective abortion. Although, India has passed legislation prohibiting prenatal diagnostic procedures for sex detection through its Pre-Conception and Pre-Natal Diagnostic Techniques (PC-PNDT) Act, 1994 (amended in 2003), the practice of gender-biased sex selection persists and is used in many societies.

However, these advanced prenatal diagnostic techniques are legally permitted to use in India to detect genetic defects, and there is a wide range of genetic diseases that are being diagnosed prenatally; there is no denying the fact of the existing misuse of this technology, which has contributed to the skewed sex ratios (29).

In the context of shrinking family sizes and reduced fertility, the desire to have at least one boy puts the pressure on parents to use prenatal sex selection as bearing a son is the primary role of the wife and a way to raise her status in her husband's family household (38).

## Social Consequences of Prenatal Sex Selection in India

The demographic repercussions of prenatal sex selection are predictable: birth imbalances result in disproportionate sex ratio cohorts that have already reached maturity. There are signs that this could have long-term social consequences, particularly in a “marriage squeeze,” leading to increased migration, bride trafficking and abduction, increasing polyandry, and forced marriages. Some villages in Haryana and Punjab in India have such poor sex ratios that men “import” brides from other States. The exploitation of these brides often accompanies this. Inability to produce a son adversely impacts the women's mental health and limits their value for making male heirs.

In addition to the previously mentioned PC-PNDT act, India has passed several legislations to decrease gender discrimination toward the girl child. To mention a few, there are (a) The Infanticide Regulation Act, 1870, (b) The Child Marriage Restraint Act 1929, amended in 2006, and (c) The Dowry Prohibition Act 1961, amended in 1985. These are well in place; however, the most pressing need is change at the societal and cultural levels toward valuing women, and this is imperative to firmly addressing pre-natal sex selection in the country.

To tear the practice of prenatal sex selection from its roots, we need to be cognizant of the co-occurrence of significant factors that favor it, such as (a) access to sex-selection technology, declining fertility, and family size preferences, (b) an unchanging patriarchal context, characterized by age and gender-stratified social systems, (c) and a continuing strong preference for sons, which has been attributed to distortions in sex ratios at birth in the country (39).

## India's Efforts Toward an Equitable Gender Environment

While addressing gender imparity, the Government of India has taken several initiatives to protect and empower the girl child. Different welfare schemes were launched at central and state levels to encourage the upliftment of the status of girls in the country. National schemes like BetiBachao and BetiPadhao were launched in 2015, focusing on eliminating gender-based abortions, ensuring girl child safety, greater well-being during infancy, and promoting girl child education. In addition, it administers to raise an understanding of the importance of the right toward property inheritance. Other schemes, such as Sukanya Samridhi Yojna, Balika Samridhi Yojna, CBSE Udaan Yojna, and National Scheme of Incentives to Girls for Secondary Education, have been implemented to enhance the school and college enrollments, increase the marriageable age, and strengthen the families to bear the marriage expenses of their girl child.

## Ethical Issues Regarding Prenatal Sex Selection

Nonetheless, the widespread sex selection leads to the disadvantaged girl child at the societal level and significantly affected sex ratios at birth. However, beyond the medical advantage of sex selection on a broader scale, there are also ethical concerns about sex selection as a choice individuals make. This includes using it as a tool for “family balancing” when the option is not primarily opted for a strong son preference.

## Conclusion

While India is consistently taking measures to enhance women's position in society and implementing policies on augmenting the value of the girl child, the combined detrimental social norms and practices still lead to a preference for a male child. This results in gender imbalances and gender bias, further leading to female feticide, infanticide, and girl child neglect. Despite available legislative provisions, medical technology is continually being misused for sex-selective abortions. Hence, it is imperative to design strategies that embrace contemplation of social determinants to address existing gender inequality. Such measures include, but are not limited to, the provision of equitable nutrition and healthcare needs, improved education policies that promote higher education for girls, equal inheritance laws for women, and, most importantly, the dialogue in the community about the provision of an equitable environment at both social and cultural levels.

More inclusive gender-based programming focusing on eliminating prenatal sex selection should be designed with community stakeholders such as civil society organizations or local community governing bodies to double the efforts to gain a substantial impact on social norms and behaviors.

## Author Contributions

NC and VD made an equal and substantial contribution to the concept and design of the work, acquisition, and review. MSa and ShN reviewed the concept and design of the work critically. MM, MSe, SA, PK, NC, VD, and SoN helped in analysis, interpretation of data, and revision. All authors contributed to the article and approved the submitted version.

## Conflict of Interest

The authors declare that the research was conducted in the absence of any commercial or financial relationships that could be construed as a potential conflict of interest.

## Publisher's Note

All claims expressed in this article are solely those of the authors and do not necessarily represent those of their affiliated organizations, or those of the publisher, the editors and the reviewers. Any product that may be evaluated in this article, or claim that may be made by its manufacturer, is not guaranteed or endorsed by the publisher.
